# Systematic Analysis of the 4-Coumarate:Coenzyme A Ligase (4CL) Related Genes and Expression Profiling during Fruit Development in the Chinese Pear

**DOI:** 10.3390/genes7100089

**Published:** 2016-10-19

**Authors:** Yunpeng Cao, Yahui Han, Dahui Li, Yi Lin, Yongping Cai

**Affiliations:** 1 School of Life Sciences, Anhui Agricultural University, Hefei 230036, China; xfcypeng@126.com (Y.C.); ldh@ahau.edu.cn (D.L.); linyi320722@163.com (Y.L.); 2 State Key Laboratory of Tea Plant Biology and Utilization, Anhui Agricultural University, Hefei 230036, China; hyahui@163.com

**Keywords:** 4-coumarate:coenzyme A ligases (4CL), acyl-coenzyme A synthetase (ACS), pear, gene family, qRT-PCR

## Abstract

In plants, 4-coumarate:coenzyme A ligases (4CLs), comprising some of the adenylate-forming enzymes, are key enzymes involved in regulating lignin metabolism and the biosynthesis of flavonoids and other secondary metabolites. Although several 4CL-related proteins were shown to play roles in secondary metabolism, no comprehensive study on 4CL-related genes in the pear and other Rosaceae species has been reported. In this study, we identified 4CL-related genes in the apple, peach, yangmei, and pear genomes using DNATOOLS software and inferred their evolutionary relationships using phylogenetic analysis, collinearity analysis, conserved motif analysis, and structure analysis. A total of 149 4CL-related genes in four Rosaceous species (pear, apple, peach, and yangmei) were identified, with 30 members in the pear. We explored the functions of several 4CL and acyl-coenzyme A synthetase (ACS) genes during the development of pear fruit by quantitative real-time PCR (qRT-PCR). We found that duplication events had occurred in the 30 4CL-related genes in the pear. These duplicated 4CL-related genes are distributed unevenly across all pear chromosomes except chromosomes 4, 8, 11, and 12. The results of this study provide a basis for further investigation of both the functions and evolutionary history of 4CL-related genes.

## 1. Introduction

The 4-coumarate:coenzyme A ligases (4CLs) play key roles in generating Coenzyme A (CoA) esters during the hydroxycinnamic acid production step of phenylpropanoid metabolism. The general phenylpropanoid pathway channels carbon flow into diverse branching pathways of secondary phenolic metabolism and generate various classes of phenolic secondary natural products, including flavonoids, lignin, suberins, and coumarins, which play important roles in plant development and environmental interactions [1]. According to the previous report by Hamberger et al. [2], 4CL genes could encode multiple isoenzymes with distinct substrate affinities that appeared to be related to specific metabolic functions. Numerous 4CL genes have been cloned from a large number of plant species, and it has been found that some of these genes were included into small gene families among the tested species. For example, there are four or five 4CL genes in poplar (*Populus*), *Arabidopsis thaliana*, maize (*Zea mays*), and rice (*Oryza sativa*) [2,3,4,5]. Moreover, the 4CL proteins in potato (*Solanum tuberosum*) and loblolly pine (*Pinus taeda* L.) are almost identical [6,7], whereas those in white poplar (*Populus tremuloides*), soybean (*Glycine max*), california poplar (*Populus trichocarpa*), and tobacco, are structurally divergent [8,9,10,11]. 

**Figure 1:**
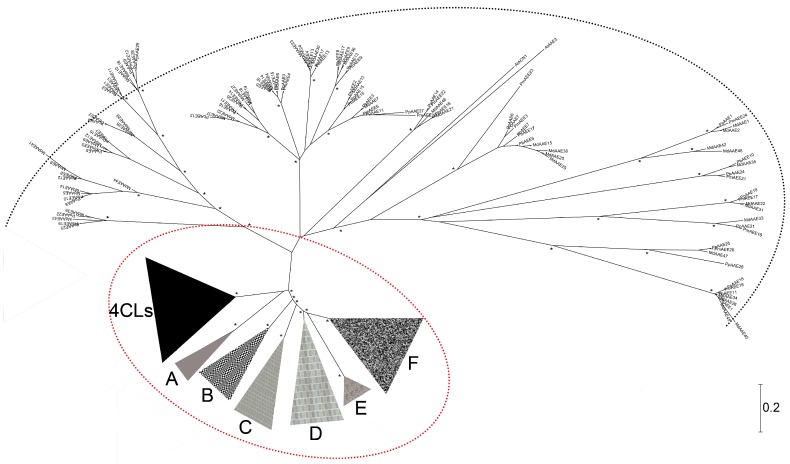
Phylogenetic relationships of 4-coumarate:coenzyme A ligase (4CL)-related proteins in *Malus x domestica*, *Prunus persica*, *Prunus mume*, *Oryza sativa*, *Arabidopsis*, and *Pyrus bretschneideri*. Asterisks represent bootstrap values of at least 70%. The large dashed arc indicates a large clade of acyl-activating enzymes (AAEs) from four Rosaceous plants (*Malus x domestica*, *Prunus persica*, *Prunus mume*, and *Pyrus bretschneideri*) as well as *Oryza sativa* and *Arabidopsis*. The red dashed ellipse indicates the clade of 4CL and acyl-coenzyme A synthetases (ACS) genes. The gene name species prefixes: Pb, *Pyrus bretschneideri*; Pp, *Prunus persica*; Pm, *Prunus mume*; Md, *Malus x domestica*; At, *Arabidopsis thaliana*; and Os, *Oryza sativa*. Gene names and models are listed in Table 1 and Table S1. The scale represents 0.2 amino acid changes.

The differential expression profiles of 4CL genes suggest that these genes, which function in the formation of different classes of benzene propane compounds, have undergone subfunctionalization [3,12]. As members of an adenylate-forming enzyme superfamily, 4CL enzymes could participate in the formation of an adenylate intermediate. According to a wealth of genome sequence data from *Malus domestica*, *Oryza sativa*, *Prunus persica*, *Pyrus bretschneideri*, *Prunus mume*, and *Arabidopsis*, some genes encoding adenylate-forming enzymes are closely related to true 4CLs, but have no known specific biochemical function. These enzymes may function in the biosynthetic pathways of various metabolic and natural products in plants. For example, based on the phylogenetic relationship of 4CLs in *Arabidopsis*, four genes with distant similarity to true 4CL genes were identified [13]. Subsequently, due to their close phylogenetic relationship to true 4CLs (bona fide 4CLs in Figure 1), eight members of a larger class of *Arabidopsis* 4CL-type adenylate-forming enzymes were classified as 4CL-like genes [14]. These 4CL-like proteins contained similar structural components, such as conserved Box I and Box II domains and a conserved substrate binding domain [15,16]. Ehlting et al. [17] identified nine genes closely related to genes encoding 4CLs, and they were annotated as 4CL or 4CL-like genes based on clade [14,18,19].

In contrast to true 4CLs, functions of 4CL-like genes are not related to flavonoid or lignin biosynthesis [14,17], suggesting that they may encode enzymes without activity towards some 4CL hydroxycinnamate substrates, such as p-coumaric, 5-hydroxyferulic, ferulic, caffeic, and sinapic acids. Indeed, seven 4CL-like recombinant proteins (GenBank no. AAO64847, AAQ56837, AEE78480, AEE29981, AED94270, AEE34025, and AEE82486) did not show catalytic activities [20]. In a subsequent analysis, several 4CL-like genes were found to encode a type of acyl-coenzyme A synthetase, which accepted both medium- and long-chain fatty acids with the cyclopentenone 12-oxo-phytodienoic acid and/or 12-oxo-phytodienoic acid derivatives as substrates under some conditions [21,22]. 12-oxo-phytodienoic acid is an intermediate for jasmonic acid biosynthesis within the octadecanoid pathway. Furthermore, β-oxidation of 12-oxo-phytodienoic acid–CoA (resulting in acyl chain shortening) led to the formation of 12-oxo-phytodienoic acidCoA thioesters via the latter steps within the pathway [23,24]. One *Arabidopsis* 4CL-like gene (GenBank no. AEE29981) was found to encode a peroxisomal 12-oxo-phytodienoic acid, a CoA ligase related to jasmonic acid biosynthesis, which has since been designated *OPCL1* [22,24]. However, to date, the biological functions of pear 4CL-like genes have not been elucidated. Based on the lack of activity of several 4CL-like enzymes against hydroxycinnamate substrates and their function as acyl-coenzyme A synthetases (ACS) to accept fatty acids, we used the name ACS to describe the products of 4CL-like genes in the pear (*Pyrus bretschneideri* Rehd.), yangmei (*Prunus mume*), peach (*Prunus persica*), and apple (*Malus x domestica*) in this report. Pears are among the most economically important fruit crops worldwide. Previous studies revealed that the stone cell content is an important factor impacting the taste of the fruit flesh in pears [25]. Pear stone cells are mainly composed of lignin [25,26]. Accordingly, to improve the quality of pear fruit, reducing the lignin content of the pear stone may be an effective method. In addition, although several 4CL-related genes that play roles in secondary metabolism have been characterized in some model species, such as rice and *Arabidopsis* [27], no study of this gene family has been conducted in pears. In the current study, we performed a genome-wide analysis of 4CL-related gene family members in the Chinese white pear [28], yangmei [29], apple [30], and peach [31] based on draft genome sequences, including gene models, phylogenetic relationships, genomic structures, chromosome locations, and other structural features. In addition, we performed quantitative real-time PCR (qRT-PCR) analysis to verify our results in pears. To the best of our knowledge, this work presented the first comprehensive study on the 4CL-related gene family in the Chinese pear. Furthermore, identification and analysis of the *Pb4CL1* gene related to lignin synthesis in the Chinese pear will help to improve the quality of pear fruit in the future.

## 2. Materials and Methods

### 2.1. Sequence Identification and Collection

The conserved protein sequences of the AMP-binding domain PF00501were obtained using the Pfam database [32]. The Hidden Markov Model (HMM) algorithm implemented in DNATOOLS software [33] was used as a query to identify all AMP-binding- domain-containing sequences in pear, peach, apple, and yangmei. A total of 156 candidate 4CL-related genes were identified. Subsequently, the SMART database [34] and Pfam database [32] were used to verify each candidate 4CL-related protein sequence as a member of the 4CL-related family, which was vital for identifying an accurate number of candidate 4CL-related proteins. Finally, a total of 149 4CL-related genes were identified in this study. According to the naming method of Clarice de Azevedo Souza et al. [35], these genes were designated *AEE*, *ACS*, and *4CL*, with species-specific identifiers used to distinguish among genes (Table 1).

A map of the chromosome locations of 4CL-related genes in pear was produced using MapInspect software (Version 1.0, Ralph van Berloo) based on their starting positions. A diagram of the exon/intron structures of these pear 4CL-related genes was generated using the online tool GSDS [36] based on genome annotation information. The conserved motifs encoded by each *Pb4CL* and *PbACS* gene were also investigated. These protein sequences were submitted to the online tool MEME (Multiple Expectation Maximization for Motif Elicitation) [37]. Parameters were set as follows: (1) optimum motif width was set to 6 and 200; and (2) maximum number of motifs was set to 20. For collinearity analysis, collinearity blocks between *Pyrus bretschneideri* Rehd. and the other species, including *Arabidopsis*, *Prunus mume*, *Prunus persica*, *Malus x domestica*, and *Oryza sativa* were downloaded from the Plant Genome Duplication Database [38] and were displayed in the collinearity map.

To analyze the promoter regions of these genes, the upstream sequences of the 4CL-related genes were analyzed based on the positions of the genes provided in the pear GigaDB database [28]. The PLACE database [39] was used to investigate putative *cis*-acting regulatory DNA elements in the promoter regions of the 4CL-related genes in the pear.

### 2.2. RNA Extraction and qRT-PCR Analysis

To investigate the expression patterns of *Pb4CL/PbACS* genes, pear fruits were sampled at 15, 39, 47, 55, 63, 79, 102, and 145 days after flowering (DAF). At least three fruits were harvested at each stage from 40-year-old pear trees grown in a horticultural field in Dangshan, Anhui, China. Total RNAs were extracted from the samples using Trizol reagent (Invitrogen, Shanghai, China) according to the manufacturer’s instructions. DNase-treated RNA was reverse-transcribed using Moloney Murine Leukemia Virus (M-MLV) reverse transcriptase (Invitrogen). Gene-specific primers for quantitative reverse-transcription PCR (qRT-PCR) were designed to generate 80–200 bp products using Beacon Designer 7 software (Applied Biosystems, Foster, CA, USA), and the *tubulin* gene [40] was used as an internal reference with primers synthesized by Sangon Biotech Co., Ltd. (Shanghai, China). The ABI7500 (Applied Biosystems) instrument was used for quantitative reverse-transcription PCR analysis of cDNA samples from different developmental stages collected from cross-pollinated varieties. The experiments were repeated three times. The relative expression levels of these genes were calculated using the 2^−ΔΔCT^ method [41]. The reactions contained the following: 10 μL of SYBR Premix Ex Taq II (2x) (Takara, Dalian, China), 1 μL of template cDNA, 0.5 μL of forward and reverse primers and free water in a final volume of 20 μL. PCR amplification was performed as follows: 50 °C for 2 min, 95 °C for 30 s, followed by 40 cycles of 95 °C for 15 s, 60 °C for 20 s, and 72 °C for 20 s.

The experiments did not involve endangered or protected species. No specific permits were required for these locations/activities because the pears used in this study were obtained from a horticultural field in Dangshan, which were demonstration orchards at Auhui Agricultural University.

### 2.3. Expression Analysis of At4CL/ACS and Os4CL/ACS Genes

Affymetrix *Arabidopsis* and *Oryza sativa* microarray data were downloaded from the ArrayExpress [42], PLEXdb [43] and Rice Oligonucleotide databases [44]. Hierarchical clustering was performed with HemI 1.0 software [45] to analyze the expression patterns of 4CL/ACS family genes in *Arabidopsis* and *Oryza sativa*.

## 3. Results and Discussion

### 3.1. The Phylogenetic Analysis of Pear 4CL-Related Genes

Using the sequence of the AMP-binding domain obtained from the Pfam database [32] as a query, we searched for 4CL-related genes from the genomic sequences of the four Rosaceae species, including the peach (*Prunus persica*) [31], apple (*Malus x domestica*) [30], yangmei (*Prunus mume*) [29], and pear (*Pyrus bretschneideri*) [28]. As a result, 149 sequences were identified (Table 1 and Table S1). A phylogenetic tree of the 149 4CL-related proteins from the four Rosaceae plants was constructed, along with those of the true 4CL proteins from *Oryza sativa* and *Arabidopsis* (Figure 1). The unrooted phylogenetic tree categorized the adenylate-forming proteins into two general clades with high bootstrap values. One large cluster contained representatives from all six plant species analyzed (the large arc in Figure 1). These adenylate-forming enzymes with various metabolic functions are conserved among these species and have a wide range of potential applications. For example, *AtACN1* acts as an entry point to the glyoxylate cycle during seed germination [46]. 

The large clade includes both *Arabidopsis ACN1* gene (*AtCN1*) [46] and many *Arabidopsis* genes for acyl-activating enzymes (AAEs) [46]. These AAEs are related to fatty acid metabolism [19]. In this study, proteins from the peach, apple, yangmei, and pear within this clade were designated as acyl-activating enzyme-like (AAEL) (Table S1) due to their sequence similarity to *Arabidopsis AAE* gene (*AtAAE*) [46]. The other clades of adenylate-forming proteins include one group of 4CL proteins and six groups of 4CL-like ACS proteins (Figure 1; clades A–F). These ACS proteins are closely related to bona fide 4CLs, forming a sister clade to clades A–F (Figure 1; clades A–F). As shown below, each of the clade A–F contains at least one representative from our examined plant species, indicating that they might have undergone gene duplication. These genes, which are probably orthologous or paralogous gene pairs, may contribute to the expansion of ACS gene family members during evolution. Both *Arabidopsis* and *Oryza sativa* proteins represented in the bona fide 4CL clade have been described and annotated [2,3,4,48]. Together with our annotated 4CL and ACS genes from the peach, apple, yangmei, and pear, they are listed in Table 1. ACS genes of the peach and yangmei are found in all clusters of A–F, whereas ACS genes from the remaining four plants are absent in some clusters. For example, *PbACS* genes are not present in cluster A. We propose that the gain or loss of some genes over the course of evolution may have helped the plant adapt to the environment. To further clarify the function of the 4CL genes in the pear, a phylogenetic tree was constructed for 4CL genes from the six species using the neighbor-joining method. Based on previous clustering approaches [2,48], these 4CL genes are clearly clustered into two categories with strong bootstrap values, as shown in Figure S1. In addition, previous studies showed that genes clustered in class I are mainly involved in lignin biosynthesis in plants, and genes clustered in class II may participate in flavonoid biosynthesis [2,48,50,51]. Then, we presented an analysis on the 4CL orthologous and paralogous gene pairs that clustered together at the terminal branch of the phylogenetic tree, and some functional clues about these genes were obtained. Therefore, we hypothesize that *Pb4CL1* and *Pb4CL3* are primarily involved in lignin biosynthesis in the pear, whereas *Pb4CL2* and *Pb4CL3* are primarily involved in flavonoid biosynthesis.

### 3.2. Structural Analysis of Pear 4CL-Related Genes

**Figure 2:**
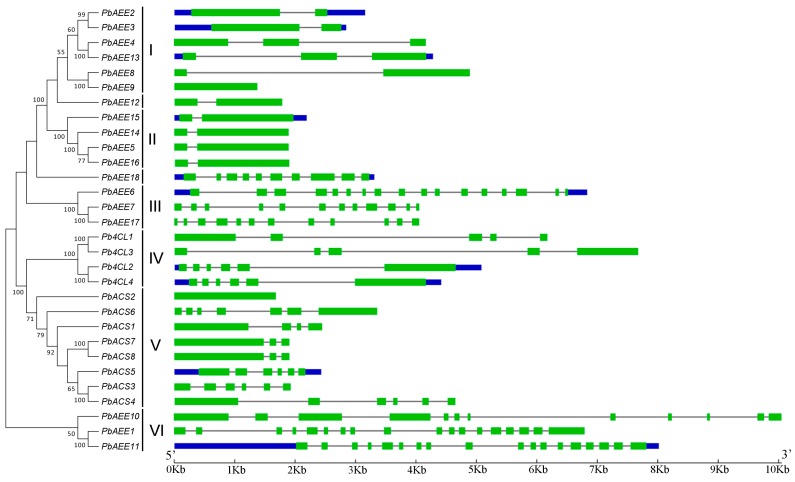
Phylogenetic and exon–intron structure analyses of the 30 predicted pear 4CL-related genes. The **neighbor joining** (NJ) tree is shown on the left. The phylogenetic tree was constructed with MEGA 6.0 [52] using the full-length amino acid sequences of the 30 pear 4CL-related proteins. Bootstrap values are represented by black numbers. The gene structures are shown on the right. Exons, introns and untranslated regions (UTRs) are indicated by green boxes, gray lines, and blue boxes, respectively. The length of each 4CL-related gene can be estimated based on the scale at the bottom.

An unrooted phylogenetic tree containing all 4CL-related genes in the pear was constructed (Figure 2) using the neighbor-joining method with bootstrap values (1000 replicates). To facilitate the research and analysis, six groups (class I–VI) were classified according to bootstrap values (>50) (Figure 2). By contrast, some 4CL-related members were beyond the six groups because of low bootstrap values (<50) of the neighbor-joining tree. As shown in Figure 2, class V contains the most 4CL-related gene members (8), followed by class I (6), while class III and VI only contains three members. Notably, the 4CL-related genes from the pear formed 10 gene pairs, which had strong bootstrap support (>99%), with the exception of *PbAEE5*/*PbAEE16*.

The gene structures of the pear 4CL-related genes were drawn by GSDS [36] by comparing the DNA sequences with their corresponding coding sequences. Observation of the gene structure in pear 4CL-related genes (Figure 2) suggested the number of introns ranged from 1 to 17, with the exception of *PbAEE9* and *PbACS2* having no introns, and *PbAEE1* and *PbAEE17* contains most introns (17) among the pear 4CL-related gene family. In addition, our analysis of exon/intron structure supports the phylogeny reconstruction. The 4CL-related genes within the same groups displayed similar exon/intron structures, particularly in terms of the number of introns, although there were some exceptions. For example, in group V, *PbACS2* lacks introns, whereas *PbACS7* and *PbACS8* contain two introns. In addition, the intron length ranges from several bases to approximately 35,500 bases, which demonstrates the high sequence diversity among members of the 4CL-related gene family.

### 3.3. Collinearity Analyses

**Figure 3:**
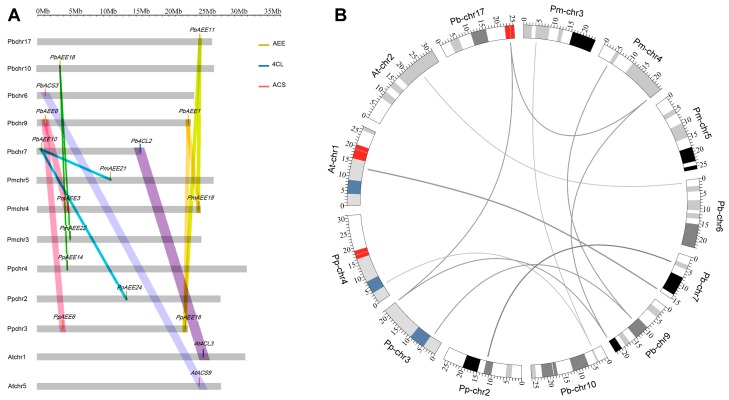
Collinearity analysis of 4CL-related regions among pear, rice, *Arabidopsis*, apple, peach, and yangmei. To identify the species of origin for each chromosome, a species identifier is included before the chromosome name: At, *Arabidopsis thaliana*; Os, *Oryza sativa*; Pb, *Pyrus bretschneideri*; Pp, *Prunus persica*; and Pm, *Prunus mume*. Different colored bars connect collinear regions between *Pyrus bretschneideri* and the other plant species.

Genomic comparison is a rapid method for transferring genomic knowledge from a well-studied group to a less-studied group. Therefore, based on their collinearity with *Arabidopsis* and *Oryza sativa* genes, we can infer the functions of 4CL-related genes in the pear to some extent. To further investigate the origin and evolutionary process of pear 4CL-related genes in Rosaceae species, we performed a comparative analysis of the genomes of the pear, peach, and yangmei. Based on the plant genome duplication event database [53], we analyzed the collinearity of 4CL-related genes of *Prunus persica*, *Pyrus bretschneideri*, *Prunus mume*, *Arabidopsis*, and *Oryza sativa* (Figure 3A,B), revealing no collinearity between the pear 4CL-related genes of rice. Remarkably, 33.3% of *PbAEE* genes share collinearity with fragments in *Arabidopsis*, *Prunus persica* and *Prunus mume*, whereas no *PbAEEs* share collinearity with fragments in rice. However, among *PbACS* and *Pb4CL* genes, we found collinear fragments only in *Arabidopsis*, for *PbACS3/AtACS9* and *Pb4CL2/At4CL3*. We did not detect collinear fragments in genes related to *PbACS*s and *Pb4CL*s in Rosaceae species such as peach and yangmei. These results suggest that the genomes of these species have undergone multiple rounds of significant chromosomal rearrangements, fusions and selective gene deletions. Due to this selective gene deletion, chromosomal colinearity blocks that may have arisen during the speciation of *Prunus persica*, *Prunus mume*, *Pyrus bretschneideri*, *Oryza sativa*, and *Arabidopsis* would be severely obscured [54].

Genes in the pear showed a closer homologous relationship with those in flowering peaches and plums belonging to the Rosaceae, as well as *Arabidopsis*, whereas they have a distant relationship with genes in rice. This is consistent with the evolution of the plant species. Several *AEE*, *4CL*, or *ACS* genes existing in *Pyrus bretschneideri*, *Prunus persica*, *Prunus mume*, and *Arabidopsis* could form orthologous gene pairs. Notably, no homologous genes of the pear 4CL-related gene family were found in the rice genome. Therefore, we postulated that pear 4CL-related genes should occur at a certain degree of specific evolution to adapt to the environment during the process of differentiation from monocotyledonous plants. In the study of the *MYB* gene family, Cao et al. found that the *AtMYB* genes in subfamily S12 may be a specific gene type to adapt to phytophagous conditions [55,56]. 

### 3.4. Genomic Distribution and Gene Duplication

**Figure 4:**
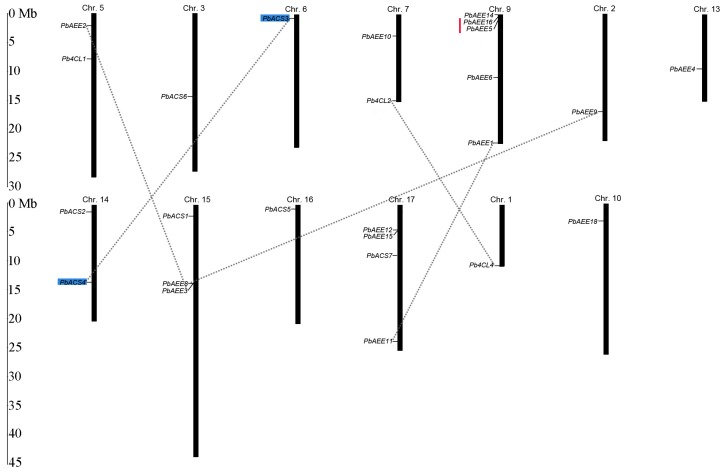
Chromosomal locations and gene duplication of 4CL-related genes in pear. Chromosome numbers are indicated at the top of each vertical black bar, the names on the left side of each chromosome correspond to the approximate locations of each 4CL-related gene. Dashed lines represent segmental duplicated genes, and the tandem duplicated gene pairs are joined by a red line. The scale is in megabases (Mb).

In almost all Rosaceae plants, one or multiple genome duplication events have occurred over the course of evolution [28,30,57]. To further investigate the effects of gene differentiation and gene duplication on 4CL-related gene families, we used MapInspect software to localize 4CL-related gene family members on the 13 pear chromosomes. As shown in Figure 4, the 4CL-related genes were found to be unevenly distributed on 13 pear chromosomes. Five gene models (*PbAEE7*, *PbAEE13*, *PbAEE17*, *PbACS8*, and *Pb4CL3*) could not be conclusively mapped to these chromosomes because they were localized to scaffolds, which may have resulted from sequencing anomalies. The number of 4CL-related genes per chromosome varies widely. Chromosome 9 contains the largest number of pear 4CL-related genes (five), followed by chromosome 17 (four). Each of chromosomes 1, 2, 3, 6, 10 and 16 has only one 4CL-related gene. Relatively high densities of 4CL-related genes were found on the tops of chromosomal regions, such as chromosome 9. According to Holub’s definition of a gene cluster [58], we found only one gene cluster (with three members) in the 4CL-related family. We propose that the members of this gene cluster underwent differentiation after chromosomal duplication. 

We also investigated gene duplication events to reveal the expansion mechanism of the 4CL-related gene family during evolution. According to Gu et al.’s definition of gene duplication events [59], we found that *PbAEE16/PbAEE5* on chromosome 9 has undergone tandem duplication, whereas nine gene pairs (*PbAEE1/PbAEE11*, *PbAEE2/PbAEE3*, *PbAEE4/PbAEE13*, *PbAEE8/PbAEE9*, *PbAEE5/PbAEE16*, *PbAEE7/PbAEE17*, *Pb4CL1/Pb4CL3*, *Pb4CL2/Pb4CL4*, and *PbACS7/PbACS8*) have undergone segmental duplication (Figure 4). Notably, among the 10 sister pairs, one gene pair (*PbACS3/PbACS4*) is located just outside the segmental duplication regions. However, their phylogenetic relationships and gene structures suggest that these genes have a similar evolutionary history, indicating that this gene pair has indeed undergone segmental duplication. We therefore assert that this gene pair has undergone gene fragment duplication (blue box logo in Figure 4) [60]. These results suggest that gene duplication events played an important role in the expansion of pear 4CL-related family members.

### 3.5. Analysis of Conserved Motifs in Pb4CL and PbACS and Their Promoter Regions

**Figure 5:**
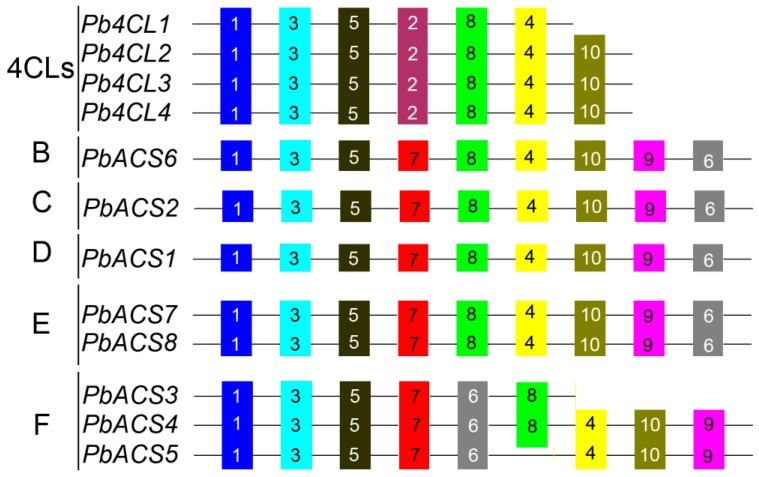
Distribution of conserved motifs in pear 4CL and ACS numbers. Motifs of 4CL and ACS proteins were identified with the Multiple Expectation Maximization for Motif Elicitation (MEME) online tool. Note that the length of each box in the proteins does not represent the actual motif size, and different motifs are indicated by different colored boxes based on results of the MEME analysis. The conserved amino acid sequences and length of each motif are shown in Table S2. Clades are defined in Figure 1.

We used the MEME online search tool to identify conserved motifs presented in 4CL/ACS proteins in the pear (Figure 5). Each motif from MEME was annotated and identified by searching the Pfam and SMART databases. Twelve sequences were categorized into two classes, which is consistent with the classification derived by the phylogenetic analysis. AMP-binding domains were represented by motifs 1, 2, 3, 4, 7, 8, 9, and 10. As shown in Figure 3, all sequences contain several motif 1-, 3-, and 5-encoding genes. Motif 7 was found in all eight *PbACS* genes, whereas Motif 2, encoding the amino acid synthesis domain, was found in all four *Pb4CL* genes. Bakolitsa et al. showed that the amino acid synthesis domain functions in amino acid synthesis [61]. We further postulate that the 4CL gene is a key gene in the phenylpropanoid pathway. Therefore, identifying an amino acid synthesis domain in the 4CL gene provides a reference for the subsequent identification of 4CL genes in other species. Furthermore, some subfamily-specific motifs with unknown functions were also identified, suggesting that these motifs are likely required for subfamily-specific functions. For example, motif 2 is specific to the 4CL subfamily. Detailed information about the conserved amino acid sequences and lengths of the 20 motifs are shown in Table S2. 

We also analyzed the promoter sequences in a 1500 bp-region upstream of the transcription start site (ATG) of the predicted *Pb4CL* and *PbACS* genes. Five regulatory elements, ABRE [62,63], LTRE [63], DRE [62], BOXP [64], and BOXL [65], were detected by searching these promoter sequences against the PLACE database [39]. Surprisingly, we found that all sequences contained putative ABRE elements in their promoter regions (Figure S2), indicating that ABA can affect the expression levels of *PbACS*s and *Pb4CL*s. Concurrently, we also found that the *Pb4CL*s and *PbACS3* genes contained BOXP and BOXL components, suggesting that these genes could be involved in the regulation of lignin biosynthesis. By comparing the distribution of the five regulatory elements (ABRE, LTRE, DRE, BOXP, and BOXL) in the promoter sequences, the four sister pairs (*Pb4CL1*/*Pb4CL3*, *Pb4CL2*/*Pb4CL4*, *PbACS3*/*PbACS4*, and *PbACS7*/*PbACS8*) were found to exhibit significant differences in their promoter regions, suggesting that the duplicated genes may not have some regulatory features in common, but may instead function in similar regulatory pathways. For example, each gene in these duplicated gene pairs contains at least one ABRE element in its promoter sequence.

### 3.6. Expression of 4CLs and ACSs during Ripening in Pear Fruit

**Figure 6:**
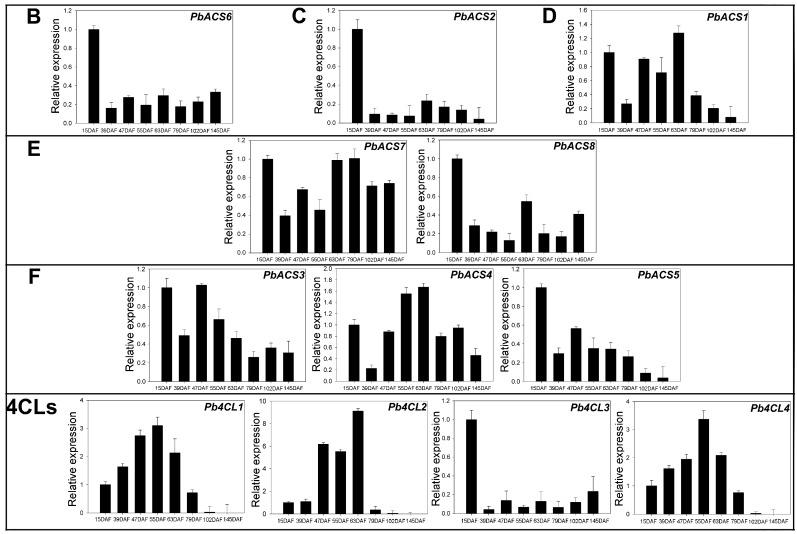
Expression levels of *Pb4CL*/*PbACS* genes during the eight stages of pear fruit development at 15 days after flowering (DAF), 39 DAF, 47 DAF, 55 DAF, 63 DAF, 79 DAF, 102 DAF and 145 DAF. These expression patterns were obtained using qRT-PCR, and the relative expression was log2 transformed. B: clade B genes; C: clade C genes; D: clade D genes; E: clade E genes; F: clade F genes; and 4CLs: 4CL genes. Clades are defined in Figure 1.

For the 4CL/ACS genes, we were interested in which ones play important roles during the development of pear fruit. In the present study, qRT-PCR analysis of pear 4CL/ACS genes revealed that these genes exhibited diverse expression patterns at 15, 39, 47, 55, 63, 79, 102, and 145 DAF (Figure 6). This result indicated that these expressed genes are functionally active, with them being expressed in several or all eight stages during the fruit development of the pear (Figure 6). The expressions of *PbACS2* and *PbACS6* in clade B, and *Pb4CL3* in bona fide 4CLs were significantly increased at 15 DAF (Figure 6), implying that these genes might play important roles in the early stage of pear fruit development. Expression of *Pb4CL1*, *Pb4CL2*, *Pb4CL4*, and *PbACS1* was obviously increased at 55 DAF, with consistent change trends of lignin content in pear fruit [25,26], indicating that these genes might be involved in the regulation of the lignin synthesis of pear fruit.

The 4CLs were shown to be involved in regulating the biosynthesis of metabolites, such as lignin and flavonoids. To help identify the *Pb4CL*s and *PbACS*s that participate in the biosynthesis of lignin or flavonoids in pear fruit during development, a composite phylogenetic tree of 4CL genes in the six species was constructed (Figure S1). The 4CLs in class I were previously shown to participate in lignin biosynthesis, whereas the 4CLs in class II function in flavonoid biosynthesis according to previous studies [2,48]. Therefore, the qRT-PCR results suggest that in the pear, only *Pb4CL1* is involved in lignin biosynthesis, whereas *Pb4CL2* and *Pb4CL4* may participate in flavonoid biosynthesis during fruit development. We also compared the expression profiles of the four duplicated gene pairs, finding that the duplicated genes within a sister pair exhibited similar expression patterns at 15, 39, 47, 55, 63, 79, 102, and 145 DAF. Differential expression patterns between the two duplicated genes were also observed. For example, the highest level of *Pb4CL1* expression was observed at 55 DAF, whereas that of *Pb4CL3* was observed at 15 DAF.

### 3.7. Expression Profiles of Rice and Arabidopsis 4CL and ACS Genes

Gene expression patterns can provide important clues about gene function. In addition, previous studies showed that orthologous genes were more likely to share correlated expression patterns compared with non-orthologous genes [66,67]. To further understand the function of 4CL/ACS genes in pear, we used publicly available genome-wide transcript profiling data from ArrayExpress [42], PLEXdb [43] and Rice Oligonucleotide databases [44] to investigate the expression patterns of 4CL/ACS genes in *Arabidopsis* and *Oryza sativa*.

#### 3.7.1. *Arabidopsis* 4CL and ACS Genes

**Figure 7:**
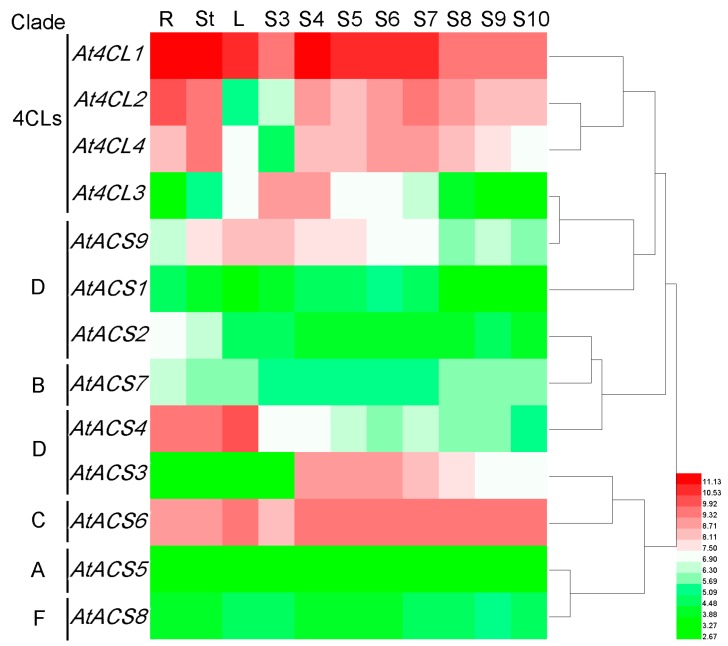
Expression patterns of ACS/4CL genes in *Arabidopsis* in different organs. R, root; St, stem; L, leaf; S3, siliques with seeds, stage 3 (mid-globular to early heart embryos); S4, siliques with seeds, stage 4 (early to late heart embryos); S5, siliques with seeds, stage 5 (late heart to mid torpedo embryos); S6, seeds, stage 6, without siliques; mid to late torpedo embryos; S7, seeds, stage 7, without siliques (late torpedo to early walking-stick embryos); S8, seeds, stage 8, without siliques (walking-stick to early curled cotyledons embryos); S9 seeds, stage 9, without siliques (curled cotyledons to early green cotyledons embryos); and S10, seeds, stage 10, without siliques (green cotyledons embryos). Clades are defined in Figure 1.

To compare the expression patterns of 4CLs and ACSs of the *Pyrus bretschneideri* with those of *Arabidopsis*, we obtained gene expression information for *Arabidopsis* in various tissues and during various stages of development from the Array Express and PLEXdb chip databases and constructed a heat map (Figure 7). During *Arabidopsis* development, the expression of *At4CL1*, *At4CL2*, and *At4CL4* increased, followed by a decrease, whereas *AtACS1*, *AtACS2*, *AtACS5*, *AtACS7*, and *AtACS8* exhibited almost no expression. Interestingly, the expression patterns of 4CLs and ACSs in *Arabidopsis* are highly similar to those of pear genes in the same cluster. For example, the expression patterns of *Pb4CL1*, *Pb4CL2*, *Pb4CL3* and *At4CL1*, *At4CL2*, and *At4CL4* were essentially the same, and the expression patterns of ACSs, such as *AtACS2* and *PbACS6* (in cluster B), were similar. However, the expression patterns of a few genes from the pear were different from those of *Arabidopsis* in the same cluster. For example, the expression of *AtACS6* (in cluster C) was relatively high during each period, whereas the expression of *PbACS2* was low beginning at 15 DAF (Figure 7).

#### 3.7.2. Rice 4CL and ACS Genes

**Figure 8:**
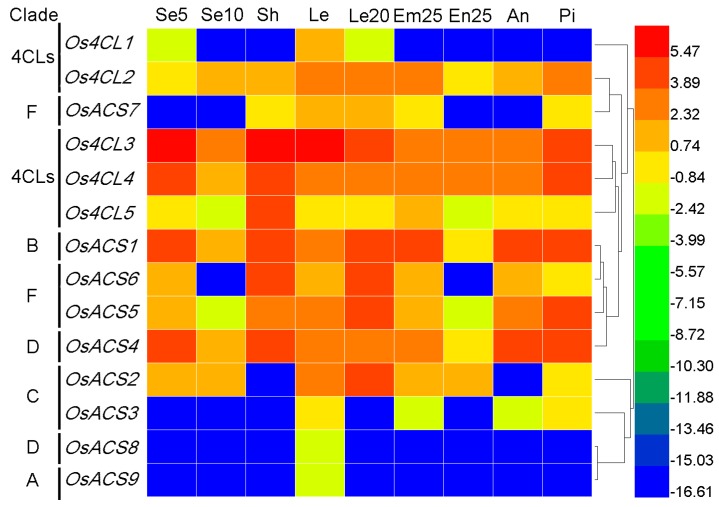
Expression patterns of ACS/4CL genes in rice in different organs. Se5, seeds at 5 DAP; Se10, seeds at 10 DAP; Sh, shoots; Le20, leaves at 20 days; Le, seedlings at the four-leaf stage; Em25, embryos at 25 DAP; En25, endosperm at 25 DAP; An, anthers; and Pi, pistils. Clades are defined in Figure 1.

We also compared the expression profiles of *Pb4CL* and *PbACS* genes in different tissues of the pear with those in rice, based on rice tissue expression microarray data from the Rice Oligonucleotide Array Databases and PLEXdb chip database and using the methods described above. The results showed that the expression level of *OsACS8* and *OsACS9* was specifically high in seedlings at the four-leaf stage (Le), instead of in other tissues (Figure 8). These different expression patterns were present in other *OsACS*s, with relatively higher levels in most tissues. For example, the expression levels of *Os4CL2*, *Os4CL3*, *Os4CL4*, and *Os4CL5* were high in various tissues over different periods (Figure 8). These results suggested that pear genes in the same cluster with rice genes could have similar expression patterns.

## 4. Conclusions

4CL enzymes participate in the regulation of lignin and flavonoid biosynthesis in plants. Proteins encoded by ACS genes may catalyze reactions involving different fatty acids or other acyl substrates. These genes are present in gene families in plants. Currently, although 4CL-related gene families were identified and characterized in several model plants (*Oryza sativa* and *Arabidopsis*), no systematic analysis of these families has previously been reported in the Rosaceae. Using DNATOOLS software, we identified 149 4CL-related genes in four Rosaceae plants, the peach, yangmei, apple, and pear. We classified these genes and analyzed their evolutionary relationships (and those of rice and *Arabidopsis*), as well as their physical locations, promoter regions, collinearity, gene structures and expression patterns using qRT-PCR analysis. The results of this study provide a characterization of the 4CL-related gene family in pear and the phylogenetic relationships of 4CL-related genes among *Prunus persica*, *Malus x domestica*, *Prunus mume*, *Oryza sativa*, *Pyrus bretschneideri*, and *Arabidopsis*. To further explore the role of these genes in the pear, we also analyzed the expression profiles of several 4CL and ACS family genes in the pear, as well as those in *Arabidopsis* and *Oryza sativa*, in various tissues and during different developmental stages. The results suggest that only *Pb4CL1* plays a key role in lignin biosynthesis and metabolic pathways in pear fruit. Our results clarify the biological function of 4CL/ACS genes in pear development and have a significant influence on our knowledge of woody plant 4CL-related genes.

## Figures and Tables

**Table 1:** *Oryza sativa*, *Arabidopsis*, *Malus x domestica*, *Prunus mume*, *Prunus persica*, and *Pyrus bretschneideri* 4-coumarate:coenzyme A ligase (4CL) and acyl-CoA synthetase (ACS) genes.

Name	Clade ^1^	Gene Model	Name	Clade ^1^	Gene Model
*Pyrus bretschneideri*			*Prunus mume*		
*Pb4CL1*	4CLs	Pbr024635.1 ^2^	*PmACS5*	D	Pm006110
*Pb4CL2*	4CLs	Pbr039972.1	*PmACS6*	F	Pm024403
*Pb4CL3*	4CLs	Pbr001283.1	*PmACS7*	F	Pm024473
*Pb4CL4*	4CLs	Pbr013445.1	*PmACS8*	A	Pm007022
*PbACS1*	D	Pbr005761.1	*PmACS9*	F	Pm024474
*PbACS2*	C	Pbr026213.1	*PmACS10*	F	Pm006911
*PbACS3*	F	Pbr027219.1	*Prunus persica*		
*PbACS4*	F	Pbr036926.1	*Pp4CL1*	4CLs	ppa003747m ^5^
*PbACS5*	F	Pbr036272.1	*Pp4CL2*	4CLs	ppa003854m
*PbACS6*	B	Pbr026415.1	*Pp4CL3*	4CLs	ppa022401m
*PbACS7*	E	Pbr040326.1	*PpACS1*	B	ppa003893m
*PbACS8*	E	Pbr041161.1	*PpACS2*	C	ppa017093m
*Malus × domestica*			*PpACS3*	C	ppa022562m
*Md4CL1*	4CLs	MDP0000260512 ^3^	*PpACS4*	D	ppa003506m
*Md4CL2*	4CLs	MDP0000295794	*PpACS5*	B	ppa003871m
*Md4CL3*	4CLs	MDP0000293578	*PpACS6*	E	ppa022057m
*Md4CL4*	4CLs	MDP0000691789	*PpACS7*	F	ppa003658m
*MdACS1*	A	MDP0000257967	*PpACS8*	F	ppa003674m
*MdACS2*	D	MDP0000716496	*PpACS9*	A	ppa003797m
*MdACS3*	E	MDP0000171033	*PpACS10*	F	ppa003742m
*MdACS4*	D	MDP0000376256	*Oryza sativa*		
*MdACS5*	E	MDP0000425292	*Os4CL1*	4CLs	Os08g14760 ^6^
*MdACS6*	E	MDP0000206055	*Os4CL2*	4CLs	Os02g46970
*MdACS7*	B	MDP0000277093	*Os4CL3*	4CLs	Os02g08100
*MdACS8*	B	MDP0000267064	*Os4CL4*	4CLs	Os06g44620
*MdACS9*	F	MDP0000179301	*Os4CL5*	4CLs	Os08g34790
*MdACS10*	F	MDP0000289629	*OsACS1*	B	Os03g05780
*MdACS11*	A	MDP0000878279	*OsACS2*	C	Os10g42800
*MdACS12*	F	MDP0000129547	*OsACS3*	C	Os08g04770
*MdACS13*	F	MDP0000262301	*OsACS4*	D	Os03g04000
*MdACS14*	F	MDP0000284973	*OsACS5*	F	Os01g67530
*MdACS15*	F	MDP0000915947	*OsACS6*	F	Os01g67540
*MdACS16*	F	MDP0000206737	*OsACS7*	F	Os07g17970
*MdACS17*	F	MDP0000671749	*OsACS8*	D	Os07g44560
*MdACS18*	F	MDP0000278716	*OsACS9*	A	Os04g24530
*MdACS19*	F	MDP0000303675	*Arabidopsis*		
*MdACS20*	B	MDP0000300934	*At4CL1*	4CLs	At1g51680 ^7^
*MdACS21*	F	MDP0000282038	*At4CL2*	4CLs	At3g21240
*MdACS22*	F	MDP0000275090	*At4CL3*	4CLs	At1g65060
*MdACS23*	D	MDP0000249003	*At4CL4*	4CLs	At3g21230
*MdACS24*	F	MDP0000723168	*AtACS1*	D	At1g20480
*MdACS25*	A	MDP0000249364	*AtACS2*	D	At1g20490
*Prunus mume*			*AtACS3*	D	At1g20500
*Pm4CL1*	4CLs	Pm013887 ^4^	*AtACS4*	D	At1g20510
*Pm4CL2*	4CLs	Pm019600	*AtACS5*	A	At1g62940
*Pm4CL3*	4CLs	Pm008736	*AtACS6*	C	At4g05160
*PmACS1*	C	Pm021042	*AtACS7*	B	At4g19010
*PmACS2*	C	Pm026124	*AtACS8*	F	At5g38120
*PmACS3*	B	Pm026358	*AtACS9*	D	At5g63380
*PmACS4*	E	Pm017552			

^1^ Clades are defined in Figure 1; ^2^ *Pyrus bretschneideri* gene models are found in the GigaDB Genome database [28]; ^3^ Apple gene models are found in the Phytozome database [30]; ^4^ *Prunus mume* and ^5^ *Prunus persica* gene models are found in the Rosaceae Genome Database [47]; ^6^ Rice gene models are found in the JGI Genome database [35]. The 4CL gene models are from Hamberger et al. [48]; ^7^ *Arabidopsis* gene models are found in the Institute for Genome Research, TAIR database [49]. The 4CL gene models are from Souza et al. [35].

## Supplementary Materials

The following are available online at www.mdpi.com/2073-4425/7/10/89/s1, Table S1: Gene names and sequences used in Figure 1, Figure S1: Phylogenetic relationships of rice, Arabidopsis, apple, yang mei, peach and pear 4CL proteins. The tree was generated with MEGA 6.0 using the NJ method, Table S2: Major MEME motif sequences in pear 4CL/ACS proteins, Figure S2: Distribution of major DNA elements in the promoter sequences of the 12 4CL and ACS genes in pear. Putative ABRE, LTRE, DRE BOXL and BOXP core sequences are represented by different symbols, as indicated.
