# Supplementary material for: Systematic Analysis of the 4-Coumarate:Coenzyme A Ligase (4CL) Related Genes and Expression Profiling during Fruit Development in the Chinese Pear
Source: Genes (Basel). 2016 Oct 19;7(10):89. doi: 10.3390/genes7100089 (PMC5083928; doi:10.3390/genes7100089)
Supplement: Supplementary file 1 [file genes-07-00089-s001.docx]

Supplementary Materials: Systematic Analysis of the 4-Coumarate:Coenzyme A Ligase (4CL) Related Genes and Expression Profiling during Fruit Development in Chinese Pear

Yunpeng Cao, Yahui Han, Dahui Li, Yi Lin and Yongping Cai

**Table S1.** Gene names and sequences used in Figure 1.

| **Gene Name in Figure 1** | **Gene Model or Accession ^1^** | **Gene Name in Figure 1** | **Gene Model or Accession ^1^** |
| --- | --- | --- | --- |
| ***Prunus persica*** |  | ***Malus × domestica*** |  |
| *PpAAE1* | ppa004215m | *MdAAE34* | MDP0000179711 |
| *PpAAE2* | ppa002540m | *MdAAE35* | MDP0000213697 |
| *PpAAE3* | ppa004139m | *MdAAE36* | MDP0000165221 |
| *PpAAE4* | ppa002531m | *MdAAE37* | MDP0000166195 |
| *PpAAE5* | ppa002256m | *MdAAE38* | MDP0000271086 |
| *PpAAE6* | ppa005794m | *MdAAE39* | MDP0000283768 |
| *PpAAE7* | ppa005737m | *MdAAE40* | MDP0000177061 |
| *PpAAE8* | ppa002255m | *MdAAE41* | MDP0000149764 |
| *PpAAE9* | ppa002568m | *MdAAE42* | MDP0000650491 |
| *PpAAE10* | ppa002295m | *MdAAE43* | MDP0000283767 |
| *PpAAE11* | ppa021932m | *MdAAE44* | MDP0000637806 |
| *PpAAE12* | ppa002006m | *MdAAE45* | MDP0000791232 |
| *PpAAE13* | ppa003832m | *MdAAE46* | MDP0000289266 |
| *PpAAE14* | ppa003723m | *MdAAE47* | MDP0000300361 |
| *PpAAE15* | ppa003716m | *MdAAE48* | MDP0000689933 |
| *PpAAE16* | ppa003617m | ***Prunus mume*** |  |
| *PpAAE17* | ppa003055m | *Pm024047* | PmAEE1 |
| *PpAAE18* | ppa001641m | *Pm012920* | PmAEE2 |
| *PpAAE19* | ppa027071m | *Pm013494* | PmAEE3 |
| *PpAAE20* | ppa003732m | *Pm012986* | PmAEE4 |
| *PpAAE21* | ppa022828m | *Pm007958* | PmAEE5 |
| *PpAAE22* | ppa001870m | *Pm026836* | PmAEE6 |
| *PpAAE23* | ppa001880m | *Pm003338* | PmAEE7 |
| *PpAAE24* | ppa025823m | *Pm014946* | PmAEE8 |
| *PpAAE25* | ppa002029m | *Pm026842* | PmAEE9 |
| *PpAAE26* | ppa002031m | *Pm004804* | PmAEE10 |
| *PpAAE27* | ppa026161m | *Pm012960* | PmAEE11 |
| *PpAAE28* | ppa010213m | *Pm021572* | PmAEE12 |
| *PpAAE29* | ppa009382m | *Pm008298* | PmAEE13 |
| ***Malus x domestica*** |  | *Pm012962* | PmAEE14 |
| *MdAAE1* | MDP0000615956 | *Pm022856* | PmAEE15 |
| *MdAAE2* | MDP0000127923 | *Pm005830* | PmAEE16 |
| *MdAAE3* | MDP0000173687 | *Pm028170* | PmAEE17 |
| *MdAAE4* | MDP0000684928 | *Pm016229* | PmAEE18 |
| *MdAAE5* | MDP0000225163 | *Pm007744* | PmAEE19 |
| *MdAAE6* | MDP0000576682 | *Pm030237* | PmAEE20 |
| *MdAAE7* | MDP0000875405 | *Pm017325* | PmAEE21 |
| *MdAAE8* | MDP0000181835 | *Pm010520* | PmAEE22 |
| *MdAAE9* | MDP0000180222 | *Pm013496* | PmAEE23 |
| *MdAAE10* | MDP0000201853 | *Pm023842* | PmAEE24 |
| *MdAAE11* | MDP0000159448 | *Pm026839* | PmAEE25 |
| *MdAAE12* | MDP0000295671 | ***Pyrus bretschneideri*** |  |
| *MdAAE13* | MDP0000243097 | *PbAEE1* | Pbr038759.1 |
| *MdAAE14* | MDP0000653037 | *PbAEE2* | Pbr030475.1 |
| *MdAAE15* | MDP0000243706 | *PbAEE3* | Pbr027708.1 |
| *MdAAE16* | MDP0000314713 | *PbAEE4* | Pbr011975.1 |
| *MdAAE17* | MDP0000300146 | *PbAEE5* | Pbr008461.1 |
| *MdAAE18* | MDP0000423449 | *PbAEE6* | Pbr019162.1 |
| *MdAAE19* | MDP0000709799 | *PbAEE7* | Pbr028634.1 |
| *MdAAE20* | MDP0000188085 | *PbAEE8* | Pbr027704.1 |
| *MdAAE21* | MDP0000763968 | *PbAEE9* | Pbr037933.1 |
| *MdAAE22* | MDP0000201011 | *PbAEE10* | Pbr040753.1 |
| *MdAAE23* | MDP0000506720 | *PbAEE11* | Pbr021917.1 |
| *MdAAE24* | MDP0000319433 | *PbAEE12* | Pbr012658.1 |
| *MdAAE25* | MDP0000223843 | *PbAEE13* | Pbr003455.1 |
| *MdAAE26* | MDP0000303056 | *PbAEE14* | Pbr033574.1 |
| *MdAAE27* | MDP0000166116 | *PbAEE15* | Pbr012647.1 |
| *MdAAE28* | MDP0000465035 | *PbAEE16* | Pbr008460.1 |
| *MdAAE29* | MDP0000224857 | *PbAEE17* | Pbr028637.1 |
| *MdAAE30* | MDP0000697764 | *PbAEE18* | Pbr037490.1 |
| *MdAAE31* | MDP0000203847 | ***Arabidopsis*** |  |
| *MdAAE32* | MDP0000458882 | *AtAAE3* | At3g48990 |
| *MdAAE33* | MDP0000614535 | *AtACN1* | At3g16910 |

^1^ Sources of genes: *Pyrus bretschneideri* gene models at the GigaDB Genome database [1]. Apple gene models at the Phytozome database [2]. *Prunus mume* and *Prunus persica* gene models at the Rosaceae Genome Database [3]. *Arabidopsis* gene models at the institute for Genome Research, TAIR database [4]. 4CL gene models previously published in Clarice de Azevedo Souza et al. [5].

**Table S2.** Major Multiple Expectation Maximization for Motif Elicitation (MEME) motif sequences in pear 4CL/ACS proteins.

| **Motif No.** | **Width** | **Conserved Amino Acid Sequences** |
| --- | --- | --- |
| 1 | 132 | GSCGMLVRNMEAKIVDPETGEALPPNQQGEIWIRGPQIMKGYLNNPEATAETIDKNGWLHTGDICYFDEDGFLFIVDRIKELIKYKGYQVPPAELEAMLISHPNIADAAVIPYPDEEAGQIPMAYVVRSNGS |
| 2 | 200 | NCRHFSELSEADENDIPDVKIYPEDPVALPYSSGTTGLPKGVMLTHKGLVTSVAQQVDGENPNLYFHEDDVILCVLPLFHIYSLNSVFLCGLRVGAGILIMHKFEIGKLLELIQKYKVSIAPFVPPIVIAIAKNPMVDRYDLSSIRMVMSGAAPMGKELEDTVKAKVPNAKLGQGYGMTEAGPVLSMCMAFAKEPMPIKS |
| 3 | 41 | TEEQVMQFVAKQVAPYKRIRRVHFINAIPKSPAGKILRKEL |
| 4 | 40 | LGVLNIGAIVTTANPFYTEAEIAKQVKDSNAKLIITQPQY |
| 5 | 29 | QDDTCAILYSSGTTGTYKGVVLTHRNFIA |
| 6 | 43 | KYDLSSLRWVGCGGAPLSKEVIDRFRERFPWVEIRQGYGMTET |
| 7 | 41 | GNTIVWMQRFDFNTMLRAIERHKVSHMPVVPPIIVALVKYA |
| 8 | 21 | MGIHKGDVVMLLSPNCIEFPF |
| 9 | 19 | HDVFLCTIPMFHIYGLAYF |
| 10 | 41 | CFQNRYEYEHRPCLIDGSTGESYSYEQLWHCVHQTASGLYQ |
| 11 | 41 | DDRVHIAGSTTNGFDPKTGIYHSLLNLGPRYKIPTKHNLDT |
| 12 | 41 | HKLVPTGVPTILTSRPLSGDDSLTIEQLIEGYDPIPTELMQ |
| 13 | 29 | TQKPADIPTNLMPPEINSTPQQNLTQLQP |
| 14 | 11 | HTYCFENISQF |
| 15 | 29 | PSSADGADQNNFPKLGEDFKVVTIDDPPE |
| 16 | 15 | IITMLRWTVDETSAQ |
| 17 | 21 | DFALKNDVEIMVVDSAETEED |
| 18 | 6 | MEHHHK |
| 19 | 14 | CGAATIFISDKQAK |
| 20 | 9 | QNKQQTVSR |


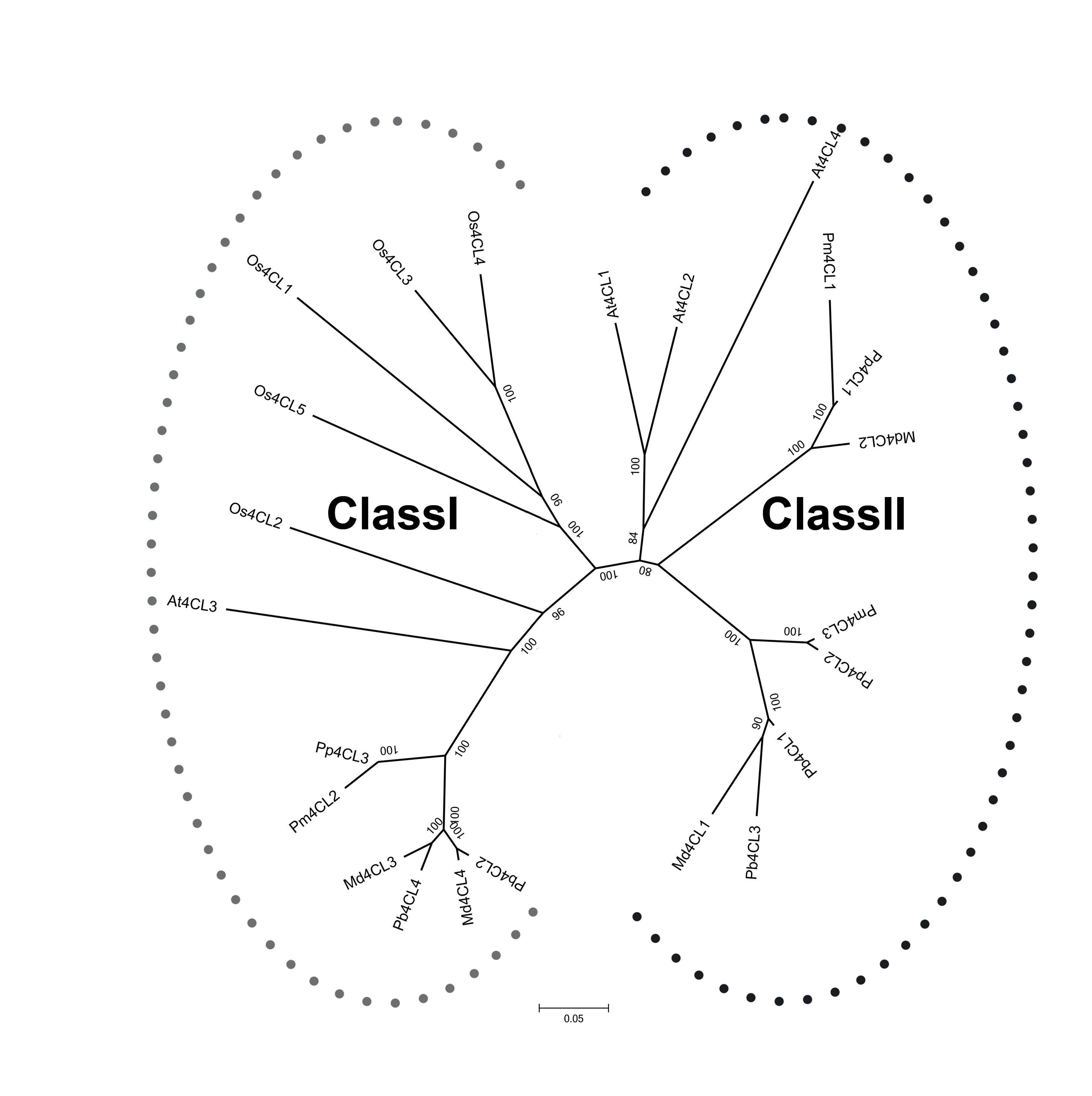


**Figure S1.** Phylogenetic relationships of rice, *Arabidopsis*, apple, yangmei, peach and pear 4CL proteins. The tree was generated with MEGA 6.0 [6] using the neighbor joining (NJ) method.


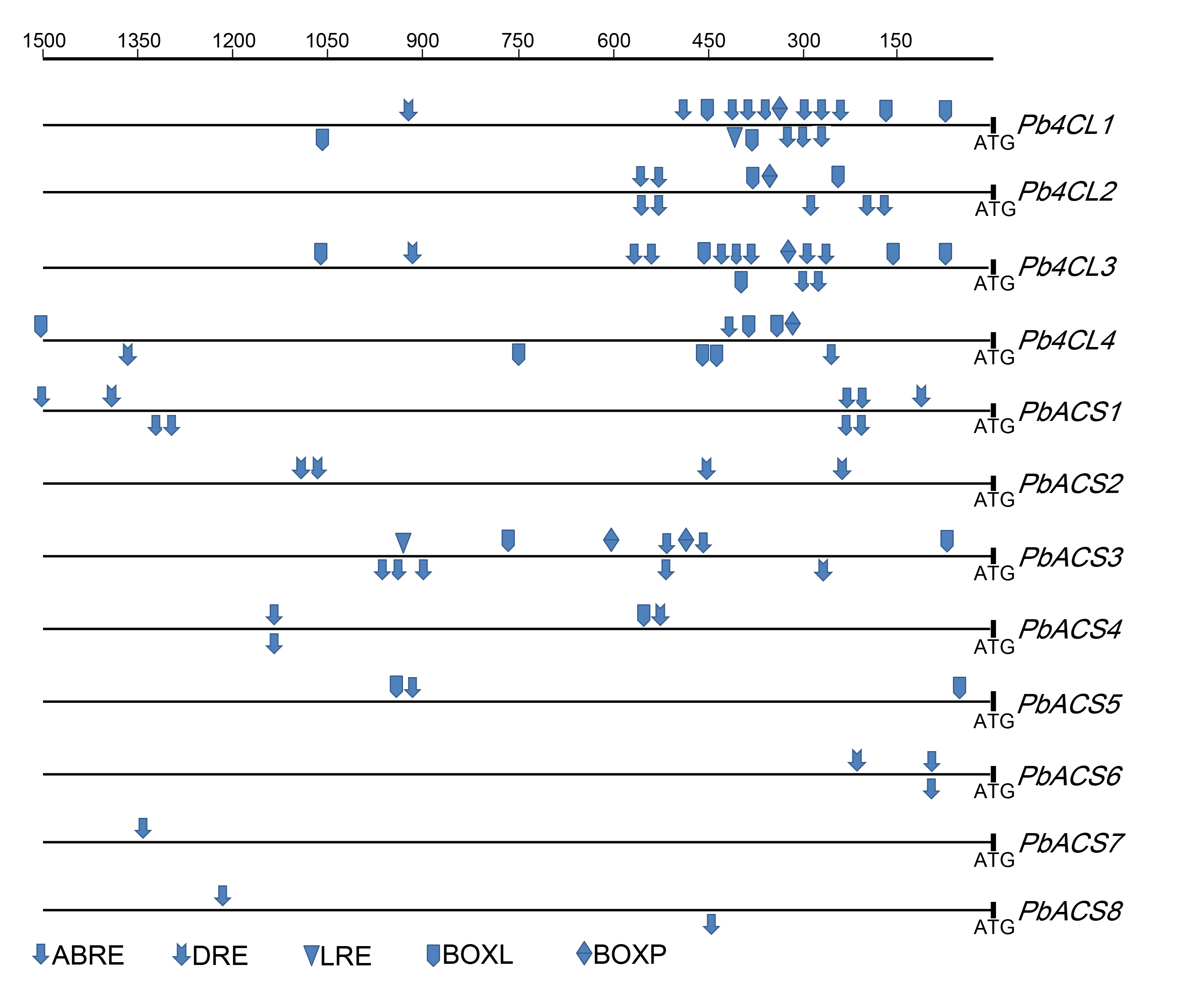


**Figure S2.** Distribution of major DNA elements in the promoter sequences of the 12 4CL and ACS genes in pear. Putative ABRE, LTRE, DRE BOXL and BOXP core sequences are represented by different symbols, as indicated.

References

1. Wu, J.; Wang, Z.; Shi, Z.; Zhang, S.; Ming, R.; Zhu, S.; Khan, M.A.; Tao, S.; Korban, S.S.; Wang, H. The genome of the pear (*Pyrus bretschneideri* Rehd.). *Genome Res.* **2013**, *23*, 396–408.
2. Velasco, R.; Zharkikh, A.; Affourtit, J.; Dhingra, A.; Cestaro, A.; Kalyanaraman, A.; Fontana, P.; Bhatnagar, S.K.; Troggio, M.; Pruss, D. The genome of the domesticated apple (*Malus* × *Domestica* Borkh.). *Nat. Genet.* **2010**, *42*, 833–839.
3. Blenda, A.; Zheng, P.; Yu, J.; Bombarely, A.; Cho, I.; Ru, S. The genome database for rosaceae (gdr): Year 10 update. *Nucleic Acids Res*. **2014**, *42*, 1237-1244.
4. Raes, J.; Rohde, A.; Christensen, J.H.; Van, d.P.Y.; Boerjan, W. Genome-wide characterization of the lignification toolbox in *Arabidopsis*. *Plant Physiol.* **2003**, *133*, 1051-1071.
5. De Azevedo Souza, C.; Barbazuk, B.; Ralph, S.G.; Bohlmann, J.; Hamberger, B.; Douglas, C.J. Genome-wide analysis of a land plant-specific acyl:coenzyme A synthetase (ACS) gene family in *Arabidopsis*, poplar, rice and *Physcomitrella*. *New Phytol.* **2008**, *179*, 987–1003.
6. Tamura, K.; Stecher, G.; Peterson, D.; Filipski, A.; Kumar, S. Mega6: Molecular Evolutionary Genetics Analysis Version 6.0. *Mol. Biol. Evol.* **2013**, 30, 2725-2729.
